# Hypertension Types and Associated Cardiovascular Risk Factors in Lithuanians Aged 50–54 Years

**DOI:** 10.3390/jcm14093177

**Published:** 2025-05-04

**Authors:** Vaida Šileikienė, Vilma Dženkevičiūtė, Alma Čypienė, Urtė Smailytė, Roma Puronaitė, Jolita Badarienė, Aleksandras Laucevičius, Eglė Butkevičiūtė, Petras Navickas, Egidija Rinkūnienė

**Affiliations:** 1 Faculty of Medicine, Vilnius University, LT-03101 Vilnius, Lithuania; 2 Clinic of Cardiac and Vascular Diseases, Vilnius University Hospital Santaros Klinikos, LT-08661 Vilnius, Lithuania; 3 State Research Institute Centre for Innovative Medicine, LT-08406 Vilnius, Lithuania; 4 Institute of Data Science and Digital Technologies, Faculty of Mathematics and Informatics, Vilnius University, LT-08412 Vilnius, Lithuania; 5 Department of Software Engineering, Faculty of Informatics, Kaunas University of Technology, LT-44249 Kaunas, Lithuania

**Keywords:** primary arterial hypertension, cardiovascular risk factors

## Abstract

**Background:** Hypertension is one of the most common cardiovascular risk factors worldwide. Additionally, epidemiological studies show a worryingly high prevalence of treatment-resistant hypertension. Especially concerning is the frequent co-occurrence of other cardiovascular risk factors, including dyslipidaemia, smoking, and diabetes mellitus. **Objectives**: The aim of this study is to investigate the prevalence of arterial hypertension and other cardiovascular risk factors in patients aged 50–54 years. **Methods**: A retrospective study was conducted on patients participating in the Lithuanian High Cardiovascular Risk Primary Prevention Programme. Data were collected from self-report questionnaires, laboratory tests, and clinical assessment. Hypertension was confirmed if systolic blood pressure was ≥140 mmHg and/or diastolic blood pressure was ≥90 mmHg or the patient had been previously diagnosed. **Results**: In total, 49155 patients—32018 (62.4%) women and 17137 (37.6%) men—were enrolled in this study. A total of 24549 (49.9%) patients were diagnosed with arterial hypertension. The prevalence of non-resistant primary hypertension was 45.9%, while the prevalence of resistant primary hypertension was 4.1%. The prevalence of dyslipidaemia was 92.79% in the non-resistant primary arterial hypertension group and was 94.59% in the resistant primary arterial hypertension group. The prevalence of smoking was higher in the non-resistant primary arterial hypertension group compared to patients with resistant hypertension (22.43% and 17.09%, respectively). A total of 23.06% of patients with resistant primary arterial hypertension had diabetes mellitus. **Conclusions**: The prevalence of primary arterial hypertension in middle-aged Lithuanians was high, reaching almost 50% in both sexes. Patients tended to have many cardiovascular risk factors simultaneously, with dyslipidaemia being the most common (prevalence > 90%).

## 1. Introduction

Cardiovascular diseases are the most common cause of death worldwide. Numerous cardiovascular risk factors have been identified—from non-controllable factors such as sex, ethnicity, and age to controllable factors such as unhealthy lifestyle, smoking, dyslipidaemia, and hypertension [1]. As many risk factors share their pathophysiology, at least in part, patients tend to have more than one cardiovascular risk factor. This tendency is worrying because it is known that the accumulation of several cardiovascular risk factors causes more damage than a single risk factor [2]. However, high blood pressure is a risk factor that should not be overlooked. According to the World Health Organisation, up to 8.5 million deaths are attributable to high blood pressure. Despite the proven negative effects on health, high blood pressure remains a widespread cardiovascular risk factor. In fact, one of the highest prevalence rates is reported in Central and Eastern Europe [3]. Despite its high prevalence, control rates are still low. In addition, studies also show a relatively high prevalence of treatment-resistant (truly resistant) hypertension, which is a dangerous condition that can have a detrimental effect on patients’ health [4]. However, national screening programmes such as the Lithuanian High Cardiovascular Risk Primary Prevention Programme (LitHir) not only contribute to a higher diagnosis rate but also improve patient education and enable appropriate treatment. For optimal primary prevention programmes, it is important to understand the prevalence and characteristics of various cardiovascular risk factors in the target population. Although middle-aged patients are more likely to suffer a cardiovascular event overall, it is well established that women suffer from cardiovascular disease approximately ten years later than men [5]. The aim of this study is to investigate the prevalence of arterial hypertension and other cardiovascular risk factors in patients aged 50–54 years.

## 2. Materials and Methods

### 2.1. Study Design and Population

The LitHir programme was introduced at a national level in 2006. Prior to this, approval was obtained from the Bioethics Committee. A detailed description of the study design has been published previously [6]. The programme is open to all Lithuanian citizens, and patients are enrolled in the programme at their general practitioner’s office if they meet the enrolment criteria. Data such as sex, age, medical history, and other cardiovascular risk factors (such as smoking, physical activity, diet, and family history) were collected. Physical measurements include height, weight (from which body mass index (BMI) is calculated), and waist circumference. Both heart rate and blood pressure were measured. Blood pressure was measured according to guidelines, following at least 5 min of rest while the patient is seated and an appropriate-sized cuff is placed on the dominant arm, which is held at heart level [7]. Laboratory tests included total cholesterol (TC), low-density lipoproteins (LDL-C), high-density lipoproteins (HDL-C), triglycerides (TGs), and serum glucose.

Arterial hypertension was confirmed if either systolic blood pressure was >=140 mmHg and/or diastolic blood pressure was >=90 mmHg or if it had been previously diagnosed and documented [7]. For this study, primary arterial hypertension was categorised as either non-resistant primary arterial hypertension or resistant primary arterial hypertension (defined as a failure to control hypertension despite optimal medical treatment consisting of at least three medications, including diuretics, and lifestyle changes). Patients were categorised as having non-resistant primary arterial hypertension if they did not meet the criteria for resistant primary arterial hypertension.

Dyslipidaemia was also determined according to ESC guidelines (LDL > 3 mmol/L, TC > 5 mmol/L, TG > 1.7 mmol/L, HDL < 1 mmol/L in men and <1.2 mmol/L in women). In addition, diabetes mellitus was confirmed if it had been previously diagnosed and documented. Obesity was defined by a BMI ≥ 30, while metabolic syndrome was diagnosed using the modified National Cholesterol Education Programme III criteria if at least three factors were present.

The data were collected from 2009 to 2019.

### 2.2. Statistical Analysis

The statistical analysis was carried out using R version 4.4.1 software. Mean values and standard deviations (SDs), as well as percentages, were given. The *t*-test was used to compare quantitative variables, while the chi-square test was used for categorical variables. Parametric methods were chosen considering the large sample size and based on the central limit theorem, which supports their robustness even when the assumption of normality is not strictly fulfilled. A *p*-value < 0.05 was considered statistically significant.

## 3. Results

### 3.1. General Characteristics

A total of 49,155 patients—32,018 (62.4%) women and 17,137 (37.6%) men—were included in this study. The mean age of the women in this study was 51.6 (SD ± 1.46) years, while the mean age of men in this study was 51.9 (SD ± 1.42) years (*p* < 0.005). Further general characteristics of the study population are listed in Table 1.

Diabetes was more common among men with resistant hypertension (27.6%; 95% CI: 24.2–31.2) compared to those with non-resistant hypertension (17.1%; 95% CI: 16.3–17.9). These non-overlapping confidence intervals indicate a statistically significant difference at the 95% confidence level, suggesting a stronger association between diabetes and resistant hypertension in men.

Diabetes was more prevalent among women with resistant hypertension (20.9%; 95% CI: 18.7–23.1) than those with non-resistant hypertension (12.2%; 95% CI: 11.7–12.8).

The confidence intervals do not overlap, indicating a statistically significant difference at the 95% confidence level.

Smoking was significantly less prevalent among men with resistant hypertension (30.2%; 95% CI: 26.7–33.9) compared to those with non-resistant hypertension (38.0%; 95% CI: 36.9–39.0).

Since the confidence intervals do not overlap, the difference is statistically significant at the 95% confidence level, indicating a potentially meaningful inverse association between smoking and resistant hypertension in men.

Smoking was slightly less common in women with resistant hypertension (10.8%; 95% CI: 9.16–12.5) compared to those with non-resistant hypertension (12.9%; 95% CI: 12.4–13.5).

The confidence intervals slightly overlap, but the *p* value is statistically significant; therefore, the difference may be real but small.

This is a borderline case, suggesting caution in interpretation.

Obesity was significantly more prevalent among men with resistant hypertension (66.8%; 95% CI: 63.0–70.4) compared to those with non-resistant hypertension (39.3%; 95% CI: 38.2–40.3).

The confidence intervals do not overlap, suggesting a statistically significant difference at the 95% confidence level.

Obesity was significantly more prevalent among women with resistant hypertension (71.6%; 95% CI: 69.2–74.0) compared to those with non-resistant hypertension (46.6%; 95% CI: 45.8–47.4).

The confidence intervals do not overlap, suggesting a statistically significant difference at the 95% confidence level.

Men with resistant hypertension were more likely to have low physical activity levels (61.3%; 95% CI: 57.4–65.0) compared to those with non-resistant hypertension (53.5%; 95% CI: 52.5–54.6).

The confidence intervals do not overlap, indicating a significant difference at the 95% confidence level, reinforcing the potential role of low physical activity levels.

Women with resistant hypertension had higher rates of low physical activity levels (74.1%; 95% CI: 71.7–76.4) compared to the non-resistant group (63.3%; 95% CI: 62.5–64.1).

The non-overlapping confidence intervals indicate statistical significance at the 95% confidence level, supporting a real association.

Among men, unbalanced diet was slightly more common in the resistant group (72.2%; 95% CI: 68.6–75.6) than in the non-resistant group (68.6%; 95% CI: 67.6–69.6). However, the confidence intervals overlap and the *p* value is not statistically significant at the 95% level.

Therefore, we cannot confidently say that there is a meaningful difference between the two groups regarding diet in men.

Unbalanced diet was more prevalent among women with resistant hypertension (74.1%; 95% CI: 71.7–76.4) than in the non-resistant group (63.3%; 95% CI: 62.5–64.1).

Since the confidence intervals do not overlap, the difference is statistically significant at the 95% confidence level, supporting a strong association between unbalanced diet and resistant hypertension in women.

### 3.2. Primary Arterial Hypertension

A total of 24549 (49.9%) patients—1327 women (47.9%) and 9222 men (53.8%)—were diagnosed with AH. The mean age of women with AH was 51.7 (SD ± 1.47) years, while that of men was 52.0 (SD ± 1.42) years (*p* < 0.001). In total, 16417 (66.9% of patients with hypertension) patients, of whom 10892 (71.1%) were women and 5525 (59.9%) were men, were undergoing treatment. Of the patients who received treatment, a correction of hypertension was achieved in 5033 patients (3589 (33.0%) women and 1444 (26.1%) men; *p* < 0.001). Other characteristics are shown in Table 2. The differences between the sexes were statistically significant in all categories.

### 3.3. Non-Resistant Primary Arterial Hypertension

**Figure 1:**
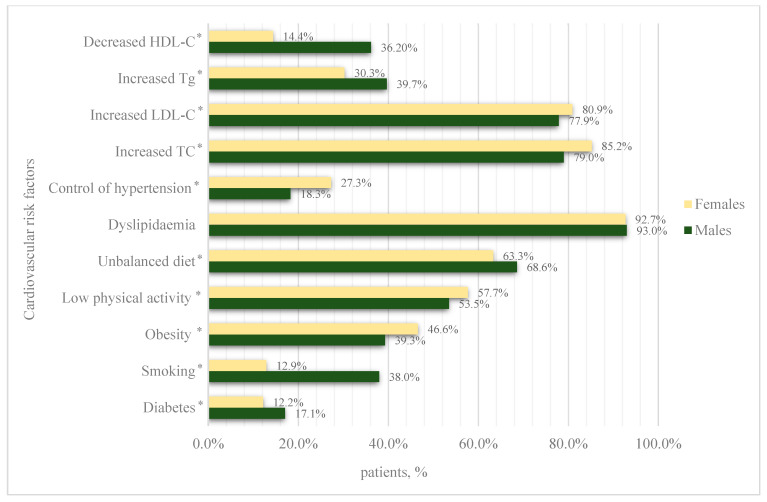
Cardiovascular risk factors among patients with non-resistant primary arterial hypertension. TC—total cholesterol; LDL-C—low-density lipoprotein cholesterol; HDL-C—high-density lipoprotein cholesterol; Tg—triglyceride. * Differences between sexes were assessed using the chi-square test (*p*-value < 0.05).

A total of 22554 patients (45.9%), of whom 13980 (43.7%) were female and 8574 (50.0%) were male, were diagnosed with non-resistant primary arterial hypertension. A correction of hypertension was achieved in 5390 patients (3819 (27.3%) women and 1571 (18.3%) men; *p* < 0.001). In total, 3181 (14.10%) patients in this group were diagnosed with diabetes. A total of 5059 (22.43%) were smokers, while 9884 (43.82%) were classified as obese. The prevalence of low levels of physical activity in this group was 56.15% (N = 12663), while the prevalence of an unbalanced diet was 65.35% (N = 14739). Overall, 20928 (92.79%) patients had dyslipidaemia. The distribution of cardiovascular risk factors by sex is shown in Figure 1.

### 3.4. Resistant Hypertension

**Figure 2:**
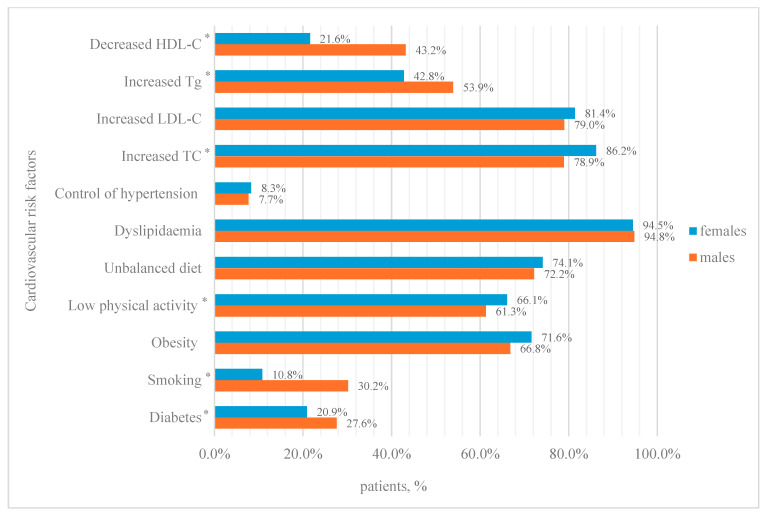
Cardiovascular risk factors among patients with primary resistant arterial hypertension. TC—total cholesterol; LDL-C—low-density lipoprotein cholesterol; HDL-C—high-density lipoprotein cholesterol; Tg—triglyceride. * Differences between sexes were assessed using the chi-square test (*p*-value < 0.05).

A total of 1995 patients (4.1%), of whom 1347 (4.2%) were women and 648 (3.8%) were men, were diagnosed with resistant hypertension. The mean age was 51.9 (+1.45) for women and 52.0 (+1.42) for men. All patients with resistant hypertension were undergoing treatment. In total, 162 patients (112 (69.1%) women and 50 (30.9%) men; *p* = 0.711) had achieved hypertension control. The prevalence of diabetes in this group was 23.06% (N = 460). Additionally, 341 (17.09%) patients reported smoking, while 1398 (70.08%) were overweight. Furthermore, 1287 (64.51%) of the patients in this group reported low physical activity levels and 1466 (73.48%) had an unbalanced diet. A total of 94.59% (N = 1887) of the patients had dyslipidaemia. The distribution of cardiovascular risk factors by sex is shown in Figure 2.

## 4. Discussion

The global prevalence of hypertension varies by region; however, it is known that some of the highest prevalence rates have been recorded in Eastern Central Europe. Lithuania has also been shown to have a high prevalence of hypertension—according to previous studies, it could be between 52 and 59% [8,9]. In this study, the prevalence of arterial hypertension was 49.9% in patients aged 50–54 years and was significantly higher in men (53.8%) than in women (47.9%). Sex differences in relation to the prevalence of hypertension have also been found in previous studies; although more men suffer from hypertension from pubertal age, its prevalence in women increases in the post-menopausal period due to hormonal changes [10]. In this study, the prevalence of non-resistant hypertension was 45.9%. The control of non-resistant hypertension was achieved in 27.3% of women and 18.3% of men. Based on the results of this study, it could be argued that control rates for hypertension are low, considering that more than half of the patients with this type of hypertension are being treated. However, these results are consistent with other research. For example, a pooled analysis showed that Eastern European regions tend to have high treatment and low control rates, indicating the gap in optimal treatment availability, patient awareness, and the remaining lack of reach of primary prevention programmes [3].

The most common risk factor in this study in both patients with resistant (94.8% women and 94.5% men) and non-resistant hypertension was dyslipidaemia (non-resistant hypertension in women is 92.7% and in men is 93.0%). Although this is to be expected, it is nevertheless a worrying observation, as these two risk factors cause endothelial dysfunction, and their combination results in a faster progression of atherosclerosis [11]. However, similar results were achieved in other studies, e.g., Mohseni et al. report that 83.95% of hypertension patients had concomitant dyslipidaemia [12]. The prevalence of diabetes was high in the entire study population, but it was particularly high in patients with resistant hypertension. Resistant hypertension has previously been associated with type 2 diabetes, and research suggests that patients who have these risk factors simultaneously may be at a higher risk of chronic complications in diabetes [13].

Based on the data included in this study, only 66.9% of patients diagnosed with arterial hypertension were treated. Treatment rates were higher in women than in men (71.1% and 59.9%, respectively). These differences in treatment may be due to the fact that men are less aware of the disease compared to women, although sex differences in hypertension awareness vary by region [10]. It is also known that male sex is associated with a lower uptake of help for a range of health problems—particularly mental health and primary care. The results of a study by Glasser et al. show that higher levels of stereotypical male gender expression are associated with lower rates of reported diagnoses and treatment for cardiovascular risk factors [14]. Another possible reason for the lower treatment rates in men could be the negative attitudes towards antihypertensive medications, as their side effects include worsening erectile function [15]. This is also consistent with the fact that women participated more actively in the programme than men.

It is proven that hypertension contributes significantly to the development of both atherosclerotic cardiovascular disease and heart failure. Hence, treatment-resistant hypertension is a devastating condition. Based on the results from the JAMP study, both heart failure and other cardiovascular events are more prevalent among patients with resistant hypertension than among those with controlled hypertension, which highlights the importance of this condition [16]. In this study, the prevalence of resistant hypertension was 4.1% and was higher in women than in men (4.2% and 3.8%, respectively). This prevalence is lower than expected, especially considering that even in the hypertensive group, the prevalence was 8.79% in women and 7.03% in men, while the expected global prevalence of truly resistant hypertension in patients treated for hypertension was 10.3% [4]. However, the reason for this lower prevalence remains unclear. The most common cardiovascular risk factors in patients with resistant hypertension were diabetes, obesity, unbalanced diet, and dyslipidaemia. It could be argued that resistant hypertension was associated with a higher prevalence of other cardiovascular risk factors; therefore, optimal treatment should not only focus on controlling hypertension but should also focus on controlling other cardiovascular risk factors. These findings are consistent with other studies, as resistant hypertension has been associated with obesity and diabetes [17,18].

## 5. Conclusions

1. The prevalence of primary arterial hypertension in the Lithuanian population aged 50–54 years was 49.9%, but only 66.9% of patients with hypertension were receiving treatment.
2. The prevalence of resistant primary arterial hypertension was 4.1%.
3. The most common concomitant cardiovascular risk factor in both resistant and non-resistant arterial hypertension was dyslipidaemia, with a prevalence of over 90%.

## 6. Strengths and Limitations

The main strength of this study is the sample size, which allows for representative data. Secondly, to the best of our knowledge, this study provides crucial data on cardiovascular risk factors and their prevalence in Lithuania. Lastly, this study involves the primary patient care system throughout Lithuania; hence, data were collected from multiple healthcare centres, which allows for the representation of patients and a generalisation of the achieved results.

However, there are several limitations of this study. As some of the data (e.g., patient compliance and smoking status) are based on self-reports, they could be considered less accurate. However, due to the large sample size of this study, it is unlikely that this has an impact on the results. Furthermore, the cross-sectional design of this study limits the evaluation of reasoning behind the achieved results.

## Figures and Tables

**Table 1:** General characteristics of the study population.

	Total (*N* = 49,155)	Females (*N* = 32,018)		Males (*N* = 17,137)		*p* Value
		95% CI		95% CI		
Age (mean, years)		51.6 (±1.46)	[51.6; 51.6]	51.9 (±1.42)	[51.9; 51.9]	<0.005
Diabetes (*N*, %)	4940	2716 (8.48%)	[8.18%; 8.79%]	2224 (13.0%)	[12.5%; 13.5%]	<0.001
Obesity (*N*, %)	15,727	10,697 (33.4%)	[32.9%; 33.9%]	5030 (29.4%)	[28.7%; 30.0%]	<0.001
Smoking (*N*, %)	10,768	4060 (12.7%)	[12.3%; 13.0%]	6708 (39.1%)	[38.4%; 39.9%]	<0.001
Low physical activity levels (*N*, %)	24,551	16,314 (51.0%)	[50.4%; 51.5%]	8237 (48.1%)	[47.3%; 48.8%]	<0.001
Unbalanced diet (*N*, %)	29,310	18,237 (57.0%)	[56.4%; 57.5%]	11,073 (64.6%)	[63.9%; 65.3%]	<0.001
Dyslipidaemia (*N*, %)	44,643	29,043 (90.7%)	[90.4%; 91.0%]	15,600 (91.0%)	[90.6%; 91.5%]	0.244
Increased LDL-C (*N*, %)	38,469	25,391 (79.3%)	[78.9%; 79.7%]	13,078 (76.3%)	[75.7%; 76.9%]	<0.001

LDL-C—low density lipoprotein cholesterol.

**Table 2:** Characteristics of patients with arterial hypertension.

	Females		Males		*p*-Value	
Total (*N*)	15,327	95% CI	9222	95% CI	-	
Age, mean (±SD) (years)	51.7 (±1.47)	[51.7; 51.7]	52.0 (±1.42)	[51.9; 52.0]	<0.001	
BMI, mean (±SD)	30.5 (±6.11)	[30.4; 30.6]	29.5 (±5.01)	[29.4; 29.6]	<0.001	
Waist, mean (±SD) (cm)	94.7 (±14.2)	[94.5; 95.0]	101 (±12.7)	[101; 102]	<0.001	
Systolic pressure, mean (±SD) (mmHg)	142 (±15.2)	[141; 142]	144 (±15.3)	[144; 144]	<0.001	
Diastolic pressure, mean (±SD) (mmHg)	86.9 (±9.17)	[86.7; 87.0]	88.8 (±9.47)	[88.6; 89.0]	<0.001	
Glucose, mean (±SD) (mmol/L)	5.54 (±1.24)	[5.52; 5.56]	5.84 (±1.62)	[5.81; 5.88]	<0.001	
TC, mean (±SD) (mmol/L)	6.16 (±1.18)	[6.14; 6.18]	5.94 (±1.21)	[5.91; 5.96]	<0.001	
LDL-C, mean (±SD) (mmol/L)	3.87 (±1.04)	[3.86; 3.89]	3.79 (±1.06)	[3.76; 3.81]	<0.001	
HDL-C, mean (±SD) (mmol/L)	1.61 (±0.44)	[1.60; 1.62]	1.39 (±0.45)	[1.38; 1.39]	<0.001	
	Total (N = 49,155)	Females (N = 32,018)		Males (N = 17,137)		*p* Value
			95%CI		95% CI	
Age (mean, years)		51.6 (±1.46)	[51.6; 51.6]	51.9 (±1.42)	[51.9; 51.9]	<0.005
Diabetes (*N*, %)	4940	2716 (8.48%)	[8.18%; 8.79%]	2224 (13.0%)	[12.5%; 13.5%]	<0.001
Obesity (*N*, %)	15,727	10,697 (33.4%)	[32.9%; 33.9%]	5030 (29.4%)	[28.7%; 30.0%]	<0.001
Smoking (*N*, %)	10,768	4060 (12.7%)	[12.3%; 13.0%]	6708 (39.1%)	[38.4%; 39.9%]	<0.001
Low physical activity levels (*N*, %)	24,551	16,314 (51.0%)	[50.4%; 51.5%]	8237 (48.1%)	[47.3%; 48.8%]	<0.001
Unbalanced diet (*N*, %)	29,310	18,237 (57.0%)	[56.4%; 57.5%]	11,073 (64.6%)	[63.9%; 65.3%]	<0.001
Dyslipidaemia (*N*, %)	44,643	29,043 (90.7%)	[90.4%; 91.0%]	15,600 (91.0%)	[90.6%; 91.5%]	0.244
Increased LDL-C (*N*, %)	38,469	25,391 (79.3%)	[78.9%; 79.7%]	13,078 (76.3%)	[75.7%; 76.9%]	<0.001

SD—standard deviation; TC—total cholesterol; LDL-C—low-density lipoprotein cholesterol; HDL-C—high-density lipoprotein cholesterol; Tgs—triglycerides.

## Data Availability

The raw data supporting the conclusions of this article will be made available by the authors on request.

## Abbreviations

The following abbreviations are used in this manuscript:

TC	Total cholesterol
LDL-C	Low-density lipoprotein cholesterol
HDL-C	High-density lipoprotein cholesterol
Tgs	Triglycerides
LitHir	Lithuanian High Cardiovascular Risk Primary Prevention Programme
SD	Standard deviation
BMI	Body mass index
ANOVA	One-way analysis of variance
